# Using Generic and Disease-Specific Measures to Assess Quality of Life before and after 12 Months of Hearing Implant Use: A Prospective, Longitudinal, Multicenter, Observational Clinical Study

**DOI:** 10.3390/ijerph19052503

**Published:** 2022-02-22

**Authors:** Luis Lassaletta, Miryam Calvino, Isabel Sanchez-Cuadrado, Piotr Henryk Skarzynski, Katarzyna B. Cywka, Natalia Czajka, Justyna Kutyba, Dayse Tavora-Vieira, Paul van de Heyning, Griet Mertens, Hinrich Staecker, Bryan Humphrey, Mario Zernotti, Maximo Zernotti, Astrid Magele, Marlene Ploder, Julia Speranza Zabeu

**Affiliations:** 1 Department of Otorhinolaryngology, Hospital La Paz. IdiPAZ Research Institute, 28046 Madrid, Spain; miryamcf@yahoo.com (M.C.); iscuadrado@gmail.com (I.S.-C.); 2 Biomedical Research Networking Centre on Rare Diseases (CIBERER), Institute of Health Carlos III, (CIBERER-U761), 28029 Madrid, Spain; 3 Institute of Physiology and Pathology of Hearing, World Hearing Center, 05-830 Kajetany, Poland; p.skarzynski@ifps.org.pl (P.H.S.); k.cywka@ifps.org.pl (K.B.C.); n.czajka@ifps.org.pl (N.C.); j.kutyba@ifps.org.pl (J.K.); 4 Fiona Stanley Fremantle Hospitals Group, Perth 6150, Australia; dayse.tavora@gmail.com; 5 Department of Otorhinolaryngology, Antwerp University Hospital, 2650 Antwerp, Belgium; paul@vandeheyning.com (P.v.d.H.); griet.mertens@uza.be (G.M.); 6 ENT Department, University of Kansas Medical Centre, Kansas City, KS 66160, USA; hstaecker@kumc.edu (H.S.); bhumphrey@kumc.edu (B.H.); 7 Department of Otorhinolaryngology, Sanatorio Allende de Córdoba, Córdoba 5000, Argentina; mario.zernotti@gmail.com (M.Z.); maxi.zernotti@gmail.com (M.Z.); 8 ENT Department, Universitätsklinikum St. Pölten, 3100 St. Pölten, Austria; astrid.magele@stpoelten.lknoe.at (A.M.); marlene.ploder@stpoelten.lknoe.at (M.P.); 9 Hospital de Reabilitacão de Anomalias Craniofaciais da Universidade de Sao Paulo, Campus Bauru, Bauru 17012-230, Brazil; julia.zabeu@usp.br

**Keywords:** quality of life, cochlear implant, SOUNDBRIDGE, BONEBRIDGE, patient-reported outcomes (PRO)

## Abstract

The primary objectives of this study were to evaluate the effect of hearing implant (HI) use on quality of life (QoL) and to determine which QoL measure(s) quantify QoL with greater sensitivity in users of different types of HIs. Participants were adult cochlear implant (CI), active middle ear implant (VIBRANT SOUNDBRIDGE (VSB)), or active transcutaneous bone conduction implant (the BONEBRIDGE (BB)) recipients. Generic QoL and disease-specific QoL were assessed at three intervals: pre-activation, 6 months of device use, and 12 months of device use. 169 participants completed the study (110 CI, 18VSB, and 41BB). CI users’ QoL significantly increased from 0–6 m device use on both the generic- and the disease-specific measures. On some device-specific measures, their QoL also significantly increased between 6 and 12 m device use. VSB users’ QoL significantly increased between all tested intervals with the disease-specific measure but not the generic measure. BB users’ QoL significantly increased from 0–6 m device use on both the generic- and the disease-specific measures. In sum, HI users experienced significant postoperative increases in QoL within their first 12 m of device use, especially when disease-specific measures were used. Disease-specific QoL measures appeared to be more sensitive than their generic counterparts.

## 1. Introduction

The World Health Organization defines “health” as “a state of complete physical, mental and social well-being and not merely the absence of disease or infirmity” [1]. Consequently, the measurement of health and the effects of health care on recipients must include changes in the frequency and severity of disease(s) and the improvement in quality of life (QoL) related to the health care (e.g., the treatment chosen). To this end, patient-reported outcomes (e.g., subjective QoL) have gained importance and attention [2,3], and these outcomes have become increasingly seen as an important measurement of treatment outcome [4,5]. The self-assessed health status has proven to be a powerful predictor of mortality and morbidity [6] and is one justification regarding choice of treatment and reimbursement [7,8,9].

Regarding assessing the effects of hearing implant (HI) use, speech understanding has often been considered the primary outcome measure. HI recipients/users, therefore, routinely complete speech perception tests in sound booths or in similarly carefully controlled settings. While the results of these tests give invaluable information on users’ hearing ability, they do not convey the full benefit (or lack thereof) of HI use because carefully controlled environments do not mimic the actual lived experience with a HI. Thus, while gains in speech understanding may correlate with gains in QoL [10,11], this is not always the case [12,13]. Therefore, to gauge the effect of HI use more fully, it is necessary to ask users how device use changes their QoL [14].

HI use can improve the QoL of a person with hearing loss [15,16]. Thus, to demonstrate the benefit of HI use, QoL assessments are now often part of a HI user assessment [10,17,18]. QoL questionnaires for HI candidates/users come in two basic varieties: generic and disease specific. Summerfield and colleagues concluded that more often disease-specific measures are needed to investigate the individuals’ subjective benefit of HI use [19]. Others argue that both generic and disease-specific questionnaires should be used [20]. 

The QoL benefit from HI use may depend upon the user’s severity of hearing loss, i.e., appropriately fit cochlear implant (CI) users with profound unaided hearing loss may derive more subjective benefit than hearing aid users with moderate hearing loss, despite having lower objective hearing levels [21]. Thus, the primary objectives of this prospective, longitudinal, multicenter, observational clinical study were to (1) to evaluate the effect of HI use on QoL over time and (2) to determine which QoL measure(s) quantify the known effect of hearing improvement on QoL with greater sensitivity.

The secondary objectives were to assess if: (1) the results on disease-specific QoL tests correlate with those from generic QoL tests, (2) participants are satisfied with their received audio processor, (3) user satisfaction correlates with QoL scores, and (4) speech test outcomes correlate with the QoL results.

## 2. Materials and Methods

### 2.1. Participants I: Inclusion and Exclusion Criteria

To be enrolled in the study, potential participants had to have postlingual hearing loss, be at least 10 years old (unless local regulations excluded anyone under 18 years old), have never used a HI, be willing and able to complete the study tasks, and give their informed consent.

All participants became users of a CI, an active middle ear implant (VIBRANT SOUNDBRIDGE (VSB)), or an active transcutaneous bone conduction implant (the BONEBRIDGE (BB)). Based on their treatment, participants were allocated to one subgroup (i.e., treatment group).

All study sites are represented in the authors’ affiliations on the title page of this manuscript. Study sites were selected as per their long experience with HIs, state-of-the art facilities, and willingness and availability to participate.

### 2.2. Participants II: Study Size Calculation

This study was of an exploratory nature. It was intended to enroll a minimum of 69 participants with a CI, 38 participants with a BB, and 38 participants with a VSB. The a-priori estimated sample sizes were based on following existing comparable data:

CI participants: Arnoldner et al. [22] reported that the overall Health Utility Index (HUI-3) mean score increased from 0.464 (SD ± 0.207) preoperatively to 0.611 (SD ± 0.190) postoperatively. Using these data with a power of 80% and an alpha-level of 0.05 for a one-sided Paired sample *t*-test, a minimum sample size of 13 subjects was calculated for each CI indication group. 

VSB participants: Edfeldt et al. [23] reported that the overall HUI-3 mean score increased from 0.57 (SD ± 0.20) before intervention to 0.66 (SD ± 0.23) after intervention. Using these data with a power of 80% and an alpha-level of 0.05 for a one-sided Paired sample t-test, a sample size of 38 subjects was calculated. The sample size calculations were performed with statistical software G*Power 3.1 [24].

BB participants: no comparable data for BB users exist. As the indications for a VSB are quite similar to a BB, it was intended to also include 38 subjects.

### 2.3. Assessments and Intervals

The three study intervals were: pre-activation, at 6 months of device use, and at 12 months of device use. Participants did not complete all tests at all intervals. Participants completed the questionnaires via paper and pencil. Clinical testing (speech tests and audiograms) was conducted as per the clinical routines at each center.

#### 2.3.1. Generic QoL

Generic QoL was assessed in all participants via the HUI-3 at all three intervals [25]. Scores on the HUI-3 range between 0.00 (i.e., dead) and 1.00 (i.e., in perfect health). Negative scores represent states that are considered worse than being dead. Mean differences of 0.03 are considered clinically important, and smaller differences, such as 0.01, can also be meaningful and important in some contexts [25]. To calculate the HUI-3 scores, all questions had to be answered by the study participant. Participants with missing items were not included in the analysis.

#### 2.3.2. Disease-Specific QoL

Disease-specific QoL was assessed in all participants via the Speech, Spatial, and Qualities of Hearing Scale (short version with 12 items) (SSQ_12_) at all three intervals [26] and via the Nijmegen Cochlear Implant Questionnaire (NCIQ) for participants with a CI at all intervals [27]. The SSQ_12_ consists of 12 items with a visual analogue scale between 0–10. It was used to measure hearing abilities across three subdomains: speech perception, spatial hearing, and general qualities of hearing. In this study, we analyzed the total score.

The NCIQ consists of three general domains (physical, psychological, and social) that can be split into the following nine subdomains: physical domain, basic sound perception, advanced sound perception, speech production, social domain, activity, social functioning, psychological functioning domain, and self-esteem. All 60 items in the NCIQ are answerable on a 5-point Likert scale. Before calculating final scores, the items were reversed if applicable and then transformed. 

Scores from each subdomain were calculated based on the average of these transformed values. A maximum of three missing answers (i.e., not answered or “not applicable”) per subdomain was allowed [27]. Subdomain scores were calculated according to the corrected scoring table published in 2017. The NCIQ was specifically developed for CI users, and as such, was not completed by participants with a bone conduction implant or a middle ear implant.

#### 2.3.3. Satisfaction with Audio Processor

Participants assessed their own satisfaction with their audio processor at 6 months of device use and at 12 months of device use via the Audio Processor Satisfaction Questionnaire (APSQ) [28]. The APSQ contains 15 items divided into three subscales: comfort, social life, and usability. Each item is answerable on a 5-point Likert scale transformed into a visual analogue scale in which a score of 0 indicates “does not agree at all” and a score of 10 indicates “fully agrees”. Participants may also answer “not applicable” if they feel an item does not apply to them. Scores are obtained by calculating the average score per item; thus, higher scores indicate great satisfaction.

#### 2.3.4. Audio Processor Daily Use

At each of the two post-activation intervals, participants reported to their clinician how many hours per day they used their audio processor. 

#### 2.3.5. Speech Understanding

Speech understanding was assessed at all intervals. Each clinic used the test(s) of their choice, as per their clinical routine.

### 2.4. Bias

Data of participating study sites were pooled. The selection criteria set for this study allowed enrolling a homogeneous population within the context of a clinical routine, a real-life setting that allowed a non-biased pooling of the results. All study sites followed the same agreed-upon protocol to prevent a treatment-by-center interaction.

### 2.5. Safety and Adverse Events

Safety was evaluated via adverse event reports.

### 2.6. Statistics

Descriptive statistics were used to report patient’s demographic (e.g., age and gender) and baseline characteristics (e.g., etiologies). Descriptive statistics were also used to present questionnaire results and speech test outcomes.

Inferential statistics were applied based on the formulated hypotheses for each of the three treatment groups. The Kolmogorov–Smirnov test was used to check for data distribution. General Linear Models for repeated measurements (RM ANOVAs) with time as factor were performed to test for a significant improvement in QoL over time. Depending on the data distribution, the Student’s *t*-test, or the Wilcoxon signed-rank test was used to test for a significant improvement in QoL between two visits. Statistical significance was set to *p* ≤ 0.05. To avoid the Type I error rate because of multiple comparisons, the *p*-values were adjusted using the Holm–Bonferroni correction method. Missing data were treated as missing values. Statistical analyses were performed with IBM SPSS Statistics Version 24 (IBM, Armonk, NY, USA). 

## 3. Results

### 3.1. Participants

A total of 169 of 171 enrolled participants completed the study. Two participants were withdrawn from analyses (one for not fulfilling the study criteria and one for not adhering to the major criteria of the study protocol). Of the 169 participants, 110 received a CI (55 females and 55 males), 18 received a VSB (nine females and nine males), and 41 received a BB (25 females and 16 males) (see Table 1). 

Choice of HI was based on indication. The choice of implant, array, and audio processor was related to availability and individual clinical preference. This explains why only 18 VSB recipients, rather than the planned *n* = 38, could be included. 

The type of hearing loss varied for VSB and BB recipients. Amongst VSB recipients: eight participants had mixed hearing loss, five had sensorineural hearing loss, two had conductive hearing loss, and data were missing for three participants. Amongst BB recipients: 16 participants had mixed hearing loss, 13 had sensorineural hearing loss, 11 had conductive hearing loss, and data were missing for one participant. All CI recipients had sensorineural hearing loss.

### 3.2. Assessments

#### 3.2.1. Generic QoL

##### HUI-3

**Figure 1:**
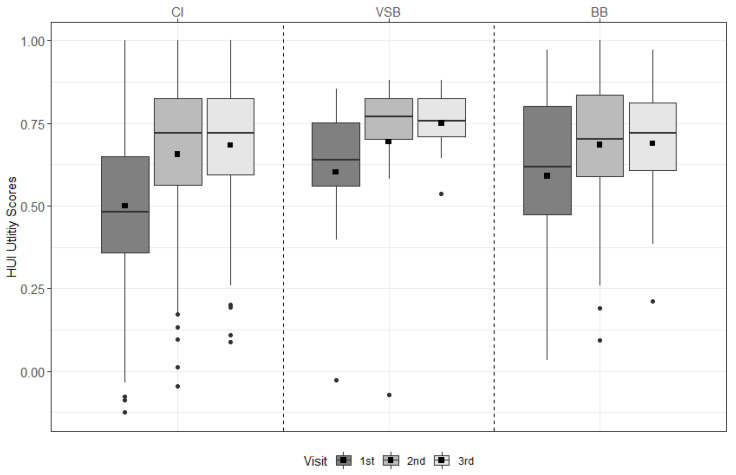
HUI-3 results across visits for each treatment group. The mean values are depicted as black squares and the medians as horizontal lines. The black circles represent outliers. Higher scores indicate better health. CI = cochlear implant, VSB = VIBRANT SOUNDBRIDGE, and BB = BONEBRIDGE.

For the CI group, scores improved significantly from pre-first fitting (pre-FF) to 6 m, from pre-FF to 12 m, and over time (all *p* < 0.001). For the BB group, scores improved significantly from pre-FF to 6 m (*p* = 0.031) and from pre-FF to 12 m (*p* = 0.018) (see Figure 1 & Supplemental Table S1).

#### 3.2.2. Disease-Specific QoL

##### SSQ_12_

**Figure 2:**
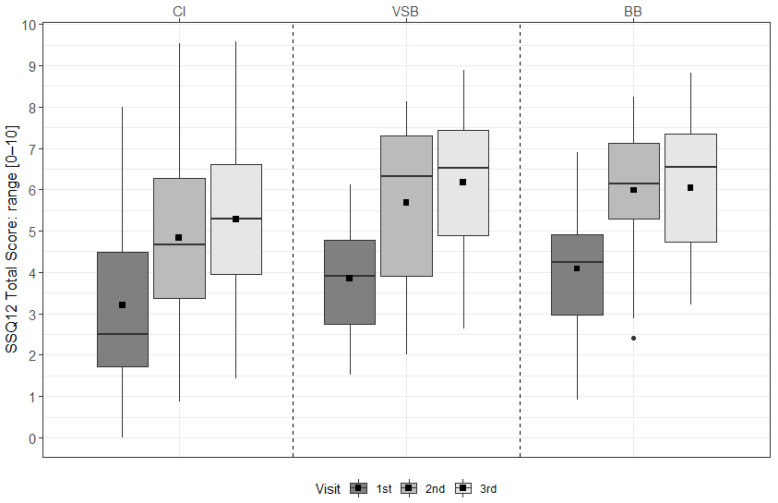
SSQ_12_ total scores across visits for each treatment group. The mean values are depicted as black squares and the medians as horizontal lines. The black circle represents an outlier. Higher scores indicate better hearing ability. CI = cochlear implant, VSB = VIBRANT SOUNDBRIDGE, and BB = BONEBRIDGE.

For the CI group, scores improved significantly from pre-FF to 6 m, from pre-FF to 12 m, from 6 m to 12 m, and over time (all *p* < 0.001). For the VSB group, scores improved significantly from pre-FF to 6 m (*p* = 0.011), from pre-FF to 12 m (*p* = 0.002), from 6 m to 12 m (*p* = 0.030), and over time (*p* < 0.001). For the BB group, scores improved significantly form pre-FF to 6 m (*p* < 0.001), from pre-FF to 12 m (*p* < 0.001), and over time (*p* < 0.001) (see Figure 2 & Table S2).

##### NCIQ

**Figure 3:**
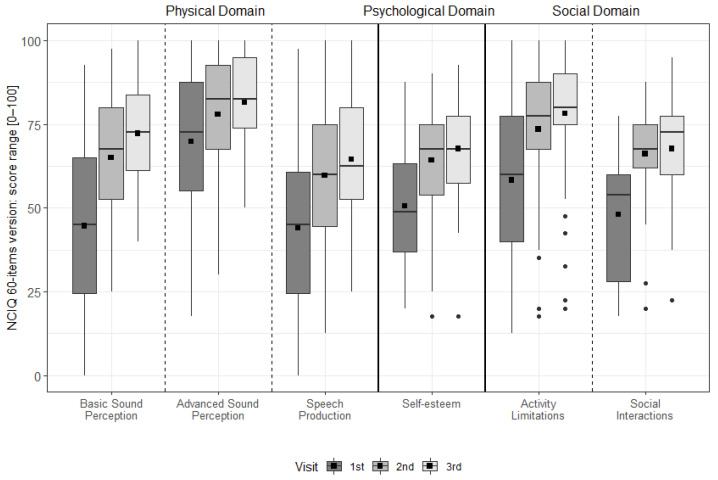
The distribution of the NCIQ domains and subdomains at each interval. The dotted vertical lines separate the domains. The mean values are depicted as black squares and the medians as horizontal lines. The black circles represent outliers. Higher scores indicate better results.

There was a significant improvement for all NCIQ subdomains from pre-FF to 6 m, from pre-FF to 12 m, and over time (all *p* < 0.001). A significant improvement was found for basic sound perception from 6 m to 12 m (*p* = 0.002). Note, as this test is designed for CI users, only the CI group was assessed (see Figure 3 & Table S3).

#### 3.2.3. Satisfaction with Audio Processor

**Figure 4:**
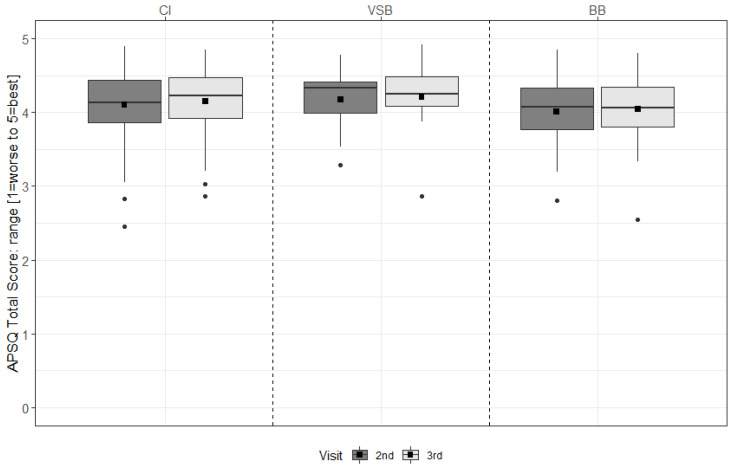
APSQ total scores of the second and the third study visit (6 m and 12 m, respectively) for each group. The mean values are depicted as black squares and the medians as horizontal lines. The black circles represent outliers. Higher scores indicate more satisfaction. CI = cochlear implant, VSB = VIBRANT SOUNDBRIDGE, and BB = BONEBRIDGE.

For the CI group, device satisfaction increased significantly from 6 m to 12 m (*p* = 0.029) (see Figure 4 & Table S4).

#### 3.2.4. Daily Use of the Audio Processor 

**Figure 5:**
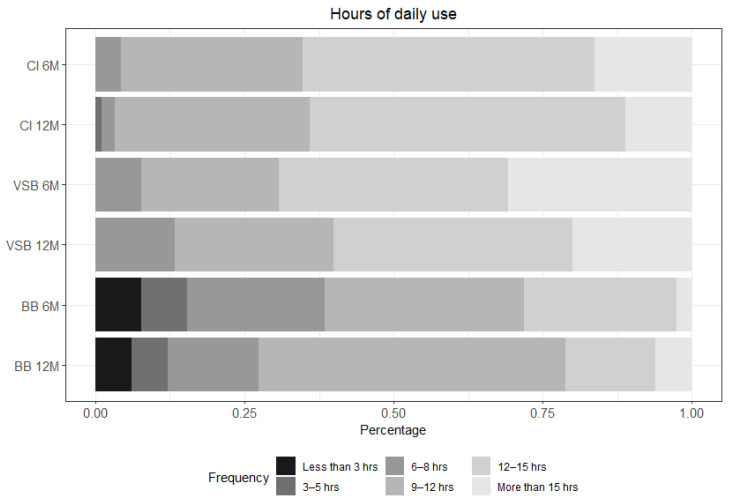
Hours of daily use for each group: CI *n* = 92 at 6 m and *n* = 89 at 12 m; VSB *n* = 13 at 6 m and *n* = 15 at 12 m; and BB *n* = 39 at 6 m and *n* = 33 at 12 m. CI = cochlear implant, VSB = VIBRANT SOUNDBRIDGE, and BB = BONEBRIDGE.

Participants were asked at both post-activation visits about their average daily use. More than 90% of the CI subjects, more than 70% of the VSB subjects, and almost 60% of the BB subjects reported an average daily use of 9 h or more after 12 months of device use (see Figure 5 & Table S5).

#### 3.2.5. Speech Understanding

Speech performance was collected according to clinical routine. The mean, the standard deviation (± SD), and the range of available speech test results in % correct and collapsed across sites were recorded. For CI users, speech performance was 25.2% ±23.0% (0–67%) at pre-FF (*n* = 19), 68.0% ±16.3% (29–96%) at 6 m (*n* = 35), and 66.8% ±21.2% (16–94%) at 12 m (*n* = 35); the pre-FF to 12 m gain was 41.6 percentage points. The Friedman test showed a significant improvement in speech performance over time for the CI group (*n* = 16; Chi-Square = 20.74; *p* < 0.001). 

Additionally, we performed pairwise comparisons with the Wilcoxon signed-rank test that showed a significant improvement from the pre-fitting (FF) visit in the best-aided condition to the 6 m visit aided with a CI (*n* = 18; *z* = −3.659; *p* < 0.001) and from the 6 m visit to the 12 m visit aided with a CI (*n* = 16; *z* = −3.409; *p* = 0.001). 

For VSB users, the median speech performance was 17.5% (0 and 35%) at pre-FF (*n* = 2), 87.5% (75 and 100%) at 6 m (*n* = 2), and 95% (range: 70–100%) at 12 m (*n* = 3); the pre-FF to 12 m gain was 70.8 percentage points. For BB users, one user scored 0 at pre-FF, and no users were tested at 6 m or 12 m. 

### 3.3. Correlations between Results

#### 3.3.1. Disease-Specific and Generic Quality of Life Measures 

For this, objective data from the 12 m visit were used. A significant correlation between the HUI-3 score and the SSQ_12_ total score and the HUI-3 and the NCIQ subdomains (all *p* < 0.001) was found for the CI group. No significant correlation between the two measures was found for the VSB or BB groups (HUI-3 and SSQ_12_).

#### 3.3.2. Quality of Life and Device Satisfaction

For this, objective data from the 12 m visit were used. No significant correlation between the APSQ total score and the HUI-3 was found in any treatment group. The correlation between the APSQ total score and the SSQ_12_ total score was significant by trend for the CI group (*p* = 0.040) and significant for the VSB group (*p* = 0.004). In CI users, a significant correlation between the APSQ total score and the NCIQ was reached in the Psychological Self-esteem subdomain (*p* = 0.002).

#### 3.3.3. Quality of Life and Speech Test Outcomes

Only data from CI users were considered because there were few speech test outcomes available for the other two groups. No significant correlations between speech test outcomes and the QoL measures (HUI-3, SSQ_12_, and NCIQ) were found.

### 3.4. Safety and Device- or Procedure-Related Adverse Events

Four device- or procedure-related adverse events were reported, one of which was severe. All events were resolved. They were as follows: two participants (RONDO and SAMBA BB) experienced pain or irritation, which was resolved by changing the magnet strength; one participant (SAMBA BB) experienced pain on the surgical side when wearing glasses, which was resolved by the participant changing his/her style of glasses; and one participant (SAMBA BB) experienced an internal device failure, which was resolved via explantation and reimplantation.

## 4. Discussion

Including QoL tests as part of the assessment battery of HI users has become common in the literature. Indeed, the role of QoL tests—and patient-reported outcomes in general—has increased so that they now play an important role in the decision making of regulatory bodies, health care payers, professionals, and users [7,8,9,29]. QoL is assessed either via a generic test or via a disease-specific test. Disease-specific tests tend to more sensitive to QoL changes after HI provision than generic tests [30]; therefore, they are often preferred in studies that do not use both generic and disease-specific assessments.

The results show that HI use can have a significant impact on users’ generic and disease-specific QoL. The results from CI users (who have severe to profound hearing loss in at least one ear) suggest that, for high levels of hearing loss, device use leads to significant improvements in QoL when assessed with disease-specific measures (NCIQ and SSQ_12_) and with a generic measure (HUI-3) (Some participants had negative HUI-3 results. Since a score of 1.00 indicates perfect health and 0.00 represents death, this should indicate that their QoL was worse than death. The explanation for this is that the multi-attribute score (the “total” score) is not the average of the single-attribute scores, rather it is calculated by a special formula provided by the HUI-3 authors, which can result in a score below zero). 

The changes in generic QoL (HUI-3) due to HI use in the CI group were relatively strong compared to the changes reported by the BB and VSB groups. A possible explanation for this could be the influence of the contralateral ear upon the user’s severity of hearing loss, i.e., appropriately fit CI users with profound unaided hearing loss may derive more subjective benefit than hearing aid users with moderate hearing loss, despite having low objective hearing levels [21]. In the CI group, many unilateral CI users with poor hearing in their contralateral ear were included in the present study. 

Improving the functionality of their implanted ear could substantially improve their QoL because it becomes their better hearing ear and the hearing in their contralateral ear is too poor to “fall back on”. In contrast, BB and VSB users were likely to have similar or even better hearing in their contralateral ear than in their implanted ear, even when best aided. Thus, the hearing benefit derived from device use would be less dramatic and, therefore, lead to smaller changes in QoL.

Interestingly, for the CI users, SSQ_12_ scores (but not HUI-3 scores) increased from the second (6 months post-FF) to the third study visit (12 months post-FF). NCIQ scores also increased significantly during this time period but only for the “Basic sound perception” subdomain. This indicates that (1) CI users derive the bigger QoL benefit within 6 m of FF and that (2) the SSQ_12_ is more sensitive, likely due to its specificity, than the generic questionnaire. 

Importantly, compared to other generic QoL measures (i.e., SF-36 or EQ-5D), the HUI-3 is more sensitive to changes in hearing; likely because the SF-36 and the EQ-5D both lack a specific hearing domain/subdomain assessment [19,31], thus, leading to differences with their sensitivity to hearing impairment [19]. The results for CI users show that the CI users experienced a real QoL benefit from CI use and that this benefit was most marked in the first 6 months of device use. After 6 months, the SSQ_12_ (and, to some extent the NCIQ) was sensitive enough to continue indicating a significant benefit, whereas the generic questionnaire was not. This could be interpreted as due to a change in quality of hearing and, potentially, in speech understanding.

This idea can be supported by the fact that the VSB but not the BB users showed a significant increase in the SSQ_12_ scores from the second to the third study visit (6 m to 12 m). Differences between these two HIs might contribute to this difference: the BB implant is screwed into the skull [32], while the VSB is an internal vibrating ossicular prosthesis [33]. This can affect postoperative healing time and means that the VSB also interacts with the middle and inner ear, while the BB does not. 

A possible result of these differences is that, for the BB, no changes in QoL are expected from the first fitting onwards. In contrast, VSB users may experience changes related to the healing and fitting process over a longer time. In turn, this may impact on hearing and secondarily on QoL. The mentioned factors could contribute to a change in quality of hearing and speech understanding, which, in turn, can be mirrored in the SSQ_12_. 

Correlational analyses between the different questionnaires used in the present study were very weak. Based on the results from this study and previously published studies, it seems recommendable to use a generic QoL measure (e.g., the HUI-3 or another questionnaire, which includes a dimension covering the senses (i.e., hearing)) and one disease-specific measurement. There are several studies that implemented this strategy and reported a high impact of using both measurements in participants with hearing loss [34,35,36]. 

In the present study, the SSQ_12_ was very sensitive to changes during the first year after FF, while the NCIQ was less sensitive: significant changes could be detected only for one subdomain. The fact that the NCIQ detected the largest changes in disease-specific QoL early after CI switch-on is in line with the results of a previous study on CI users with SSD [18]. In contrast, the SSQ_12_, which also contains several items on Quality of Hearing, was very sensitive to changes during the first year of HI use. If clinicians aim to investigate the change of QoL due to HI use in their patients, a generic QoL questionnaire can show large changes from before to after HI provision. It is important, however, to also consider that using the HUI-3 necessitates paying the questionnaire’s license fees. 

The AQOL-8D, a newer and (thus far) less-used generic QoL measure, also contains a dimension on senses, including hearing, and could be a promising free alternative [35]. Using the NCIQ would add to the generic changes with a disease-specific perspective. Data from the present study suggest that, during the first year after implantation, the SSQ_12_ can better detect smaller changes in QoL or, rather, Quality of Hearing. Another advantage of using the SSQ_12_ is that it is much shorter than the NCIQ (12 items versus 60 items). Further, unlike the SSQ_49_/SSQ_12_, there is no short version of the NCIQ, and there is no proxy version that caretakers can take in the patients’ stead. This could be an issue for some candidates/recipients. Newer questionnaires, e.g., the ERSA [37] or the CIQOL-35 [30] may also be options.

Device satisfaction, as measured with the APSQ, increased significantly during the first year of CI use. This is an interesting result because the average speech understanding results were similar for both follow-up visits. This first experience with the APSQ questionnaire in a longitudinal study suggests that this questionnaire can help clinicians evaluate user satisfaction with their device in everyday life throughout the first year of use. The APSQ could also be helpful for counselling of candidates and user rehabilitation; however, more data are needed before that can be stated with more confidence.

Regarding speech understanding results and QoL results, their lack of correlation is in line with results of previously published studies [12,14,38]. This is an important result, especially seeing as that improved speech understanding scores are generally regarded as the primary measurement of success or failure of CI use. We believe that this is outmoded and that it is very important that HI candidates and clinicians extend their view on the impact of hearing loss beyond speech understanding to include its impact on many aspects of life. 

This holistic view acknowledges that hearing loss has a broader impact on life than simply hearing and that small increases in hearing due to device use can have great benefits in a user’s life and vice versa. Thus, while speech-understanding scores are undoubtedly important, a full picture of the impact of HI use is incomplete unless users’ QoL and satisfaction with their HI are assessed before and after HI provision.

The present study has certain limitations. First, the choice of intervals means that this study could not capture changes in QoL occurring before 6 months of use or after 12 months of use. Other published studies suggested that (1) a significant increase in QoL after HI treatment can be measured already after 3 months [18] and that (2) QoL does not change after more than 12 months of HI use [10]. Secondly, CI users were not differentiated according to type of user, i.e., they contain both unilaterally and bilaterally deaf participants. This, however, enables the results to show clinicians what the broad range of CI users experience. Thirdly, one has to be cautious when interpreting the inferential statistical results of the BB group, and especially the VSB group, because of the low number of participants.

## 5. Conclusions

Improvements in QoL are an important result of HI use. The results of the present study showed that this improvement was significant within the first year of HI use, especially when evaluated using disease-specific measures. The significant increases in QoL did not correlate with increased speech perception. This finding highlights that real life benefits of HI use should not be extrapolated from in-clinic speech testing; instead, disease-specific measures are an important tool in assessing user benefit.

## Figures and Tables

**Table 1:** Age and duration of deafness for each device recipient type. Note: Variations in *n* are due to missing data.

Group	*N*	*n*	Age at Implantation (Years)	*n*	Duration of Deafness (Years)	CI	Audio Processor	Array
CI	111	98	56.7 (16.8–85.8)	79	20.3 (0–67)	SONATA = 37 SYNCHRONY = 22 SYNCHONRY PIN = 22 CONCERTO = 12 CONCERTO PIN = 5 data missing = 12	SONNET = 65 RONDO = 14 SONNET EAS = 10 OPUS 2 = 9 data missing = 12	FLEX28 = 57 FLEX24 = 23 STANDARD = 9 FLEX20 = 4 COMPRESSED = 3 MEDIUM = 1 FLEXSOFT = 1 data missing = 12
VSB	18	15	49.4 (20.0–74.2)	11	18.7 (8–40)	n/a	SAMBA = 13 Amadé = 2 data missing = 4	n/a
BB	41	40	40.2 (14.8–73.7)	25	18.4 (1–56)	n/a	SAMBA BB = 39 Amadé BB = 1 data missing = 1	n/a

CI = cochlear implant, VSB = VIBRANT SOUNDBRIDGE, and BB = BONEBRIDGE.

## Data Availability

The data presented in this study are available on reasonable request from the corresponding author.

## Supplementary Materials

The original contributions presented in the study are included in the supplementary materials. They can be downloaded at: https://www.mdpi.com/article/10.3390/ijerph19052503/s1, Table S1: HUI-3 scores for each group at each interval and all associated *p* values; Table S2: SSQ_12_ scores for each group at each interval and all associated *p* values; Table S3: NCIQ scores (mean, standard deviation, and range) for each interval and subdomain; Table S4: APSQ scores (mean, standard deviation, and range) for each group at each interval; Table S5: Self-assessed hours of daily use for each group at the 6 m and 12 m intervals.
