# Supplementary material for: Using Generic and Disease-Specific Measures to Assess Quality of Life before and after 12 Months of Hearing Implant Use: A Prospective, Longitudinal, Multicenter, Observational Clinical Study
Source: Int J Environ Res Public Health. 2022 Feb 22;19(5):2503. doi: 10.3390/ijerph19052503 (PMC8909702; doi:10.3390/ijerph19052503)
Supplement: Supplementary file 1 [file ijerph-19-02503-s001.zip › ijerph-1574816-supplementary.pdf]

Supplementary Table S1: HUI-3 scores for each group at each interval and all associated p values. Higher scores indicate better hearing health. FF = first fitting. CI = cochlear implant, VSB = VIBRANT SOUNDBRIDGE, BB = BONEBRIDGE

Test	*Device*	**Interval**					
		n	Pre-FF	n	6m	n	12m
**HUI-3**	CI	91	0.5003	86	0.6558	80	0.6837
	VSB	13	0.6030	12	0.6943	12	0.7514
	BB	39	0.5913	38	0.6854	31	0.6888

Supplementary Table S2: SSQ_{12} scores for each group at each interval and all associated p values. Higher scores indicate better hearing ability. FF = first fitting, CI = cochlear implant, VSB = VIBRANT SOUNDBRIDGE, BB = BONEBRIDGE

Test	*Device*	**Interval**					
		n	Pre-FF	n	6m	n	12m
SSQ12	CI	91	3.2015	89	4.8186	83	5.2656
	VSB	15	3.8564	13	5.6845	15	6.1737
	BB	35	4.0861	34	5.9858	30	6.0456

Supplementary Table S3: NCIQ scores (mean, standard deviation, and range) for each interval and subdomain. Higher scores indicate better results. FF = first fitting, m = month

	Interval					
	**n**	**Pre-FF**	**n**	**6m**	**n**	**12m**
**Physical domain**						
Basic sound perception	80	44.6 ± 26.1 (0.0–92.5)	73	65.0 ± 19.2 (25.0–97.5)	71	72.3 ± 16.2 (40.0–100.0)
Advanced sound perception	71	69.8 ±20.3 (17.5–100.0)	69	77.9 ±18.7 (30.0–100.0)	63	81.5 ±14.2 (50.0–100.0)
Advanced speech production	76	44.0 ±25.4 (0.0–97.5)	68	59.7 ±21.0 (12.5–100.0)	65	64.5 ±18.4 (25.0–100.0)
**Psychological domain**						
Self-esteem	76	50.6 ±18.0 (20.0–87.5)	63	64.2 ±16.5 (17.5–90.0)	67	67.7 ±14.1 (17.5–92.5)
**Social domain**						
Activity Limitations	61	58.3 ±24.1 (12.5–100.0)	58	73.5 ±20.3 (17.5–100.0)	54	78.1 ±18.5 (20.0–100.0)
Social Interactions	38	48.0 ±18.5 (17.5–77.5)	28	66.2 ±15.3 (20.0–87.5)	33	67.7 ±15.2 (22.5–95.0)

Supplementary Table S4: APSQ scores (mean, standard deviation, and range) for each group at each interval. Higher scores indicate more satisfaction. CI = cochlear implant, VSB = VIBRANT SOUNDBRIDGE, BB = BONEBRIDGE

	**Interval**			
	**N**	**6m**	**n**	**12m**
**CI**	92	4.106 ±0.5 (2.5–4.9)	87	4.153 ±0.4 (2.9–4.9)
**VSB**	13	4.178 ±0.4 (3.3–4.8)	14	4.214 ±0.5 (2.9–4.9)
**BB**	39	4.014 ±0.4 (2.8–4.9)	34	4.042 ±0.5 (2.5–4.8)

Supplementary Table S5: Self-assessed hours of daily use for each group at the 6m and 12m intervals. CI = cochlear implant, VSB = VIBRANT SOUNDBRIDGE, BB = BONEBRIDGE

	CI users				***VSB users***				***BB users***			
Interval	6m *(n=92)*		12m *(n=89)*		6m *(n=13)*		12m *(n=15)*		6m *(n=39)*		12m *(n=33)*	
	**n**	**%**	**n**	**%**	**n**	**%**	**n**	**%**	**n**	**%**	**n**	**%**
Less than 3 hrs	0	0	0	0	0	0	0	0	3	7.7	2	6.1
3-5 hrs	0	0	1	1.1	0	0	0	0	3	7.7	2	6.1
6-8 hrs	4	4.3	2	2.2	1	7.7	2	13.3	9	23.1	5	15.2
9-12 hrs	28	30.4	29	32.6	3	23.1	4	26.7	13	33.3	17	51.6
12-15 hrs	45	48.9	47	52.8	5	38.5	6	40.0	10	25.6	5	15.2
More than 15 hrs	15	16.3	10	11.2	4	30.8	3	20.0	1	2.6	2	6.1
